# From complex data to biological insight: ‘DEKER’ feature selection and network inference

**DOI:** 10.1007/s10928-021-09792-7

**Published:** 2021-11-17

**Authors:** Sean M. S. Hayes, Jeffrey R. Sachs, Carolyn R. Cho

**Affiliations:** Quantitative Pharmacology and Pharmacometrics, Merck & Co., Inc., Kenilworth, NJ USA

**Keywords:** Network inference, Feature selection, Machine learning, Multiomics, Systems biology

## Abstract

Network inference is a valuable approach for gaining mechanistic insight from high-dimensional biological data. Existing methods for network inference focus on ranking all possible relations (edges) among all measured quantities such as genes, proteins, metabolites (features) observed, which yields a dense network that is challenging to interpret. Identifying a sparse, interpretable network using these methods thus requires an error-prone thresholding step which compromises their performance. In this article we propose a new method, DEKER-NET, that addresses this limitation by directly identifying a sparse, interpretable network without thresholding, improving real-world performance. DEKER-NET uses a novel machine learning method for feature selection in an iterative framework for network inference. DEKER-NET is extremely flexible, handling linear and nonlinear relations while making no assumptions about the underlying distribution of data, and is suitable for categorical or continuous variables. We test our method on the Dialogue for Reverse Engineering Assessments and Methods (DREAM) challenge data, demonstrating that it can directly identify sparse, interpretable networks without thresholding while maintaining performance comparable to the hypothetical best-case thresholded network of other methods.

**Supplementary Information:**

The online version contains supplementary material available at 10.1007/s10928-021-09792-7.

## Introduction

In recent years the availability of high-dimensional biological data has rapidly outpaced the tools available to understand it. Here, we describe an algorithm for analyzing such data using an embedded feature selection method to infer networks of mechanistic biological relationships. These networks summarize the totality of the information available in the data, and can be used to help answer many of the critical questions across drug discovery and development: which biomarkers and targets to select, what differences in biology between pre-clinical species exist, what is a drug’s mechanism of action, and how do we identify the best combinations of treatments?

The best performing network inference methods have a significant liability, however, which we address with our new approach. These methods, such as GENIE3 (the best performing method in the ‘Dialogue for Reverse Engineering Assessments and Methods’ initiative’s challenges), do not explicitly infer networks but rather rank all possible relationships. We refer to these methods as ‘weight-focused.’ To define a network from the ranked lists produced by these weight-focused methods, the user must identify *subjectively* an unknown threshold to exclude some number of lowest ranked edges to define a final network. A common approach in the literature is to take the top million relationships, which depending on the number of features in the dataset represents anywhere from the top 0.2 to 2% of ranked relationships [8, 14, 27, 34]. The basis for selecting these thresholding values has not been well established or evaluated in benchmarking exercises such as DREAM, but has a dramatic effect on the outcome of the analysis. A high threshold risks omitting informative relationships in the data, a low threshold risks including too many errors and making meaningful interpretation impossible, and little to no information exists defining what is a ‘high’ or ‘low’ threshold for any given data type or application. Our method uses a different approach to feature selection and network inference to avoid this hand-tuned thresholding step, instead identifying the subset of relationships to form a network directly.

The network inference algorithm we describe here follows the iterative feature selection approach used by other high-performing network inference methods [10, 16], which is a natural extension of the general feature selection approach often used to analyze high-dimensional data. All feature selection approaches answer a common question: which features are meaningful predictors of a specified response? This is particularly critical for high-dimensional data, such as multi-omics datasets which combine measurements from many biological domains and analytical technologies (’omes). These datasets commonly measure thousands to hundreds of thousands of independent biological features, introducing the ‘curse of dimensionality’ [18] and violating the assumption that there are many more samples than measured quantities: this is central to many common statistical approaches [17]. Feature selection enables interpretation of high-dimensional data by identifying a subset of features which is most useful for predicting the response (predictors), thereby reducing the dimensions of the data to a scale that can be handled more easily.

Network inference can be done by reapplying this general approach of feature selection targeting each feature of the dataset separately as a response to be predicted. Through this process not only are the meaningful predictors of a response identified, but the meaningful predictors of meaningful predictors, and so on. The results of this iterative feature selection process are visualized as a network, with a predictive relationship between a selected feature and response represented as a connection (edge) between features (vertices). The network approach thus provides the same answers as a general feature selection approach focused on a specific response of interest would, but also provides additional information critical to understanding the wider biological context.

To illustrate the utility that network inference adds, consider an analysis in which a single protein is found to predict an important clinical endpoint. This information alone suggests the protein might be a good target to modulate the clinical endpoint. However, this analysis does not describe what biological features may be acting upon the prospective protein target. Homeostatic control mechanisms are a central feature of biology, and without identifying and inhibiting these control mechanisms the single protein target might be impossible to influence. If a network inference approach is used instead, all the upstream control mechanisms can be considered when making the choice of target or biomarker, avoiding costly mistakes due to unknown biology. This wider context also provides additional choices of target or biomarker, which may be easier to drug or measure, or may identify a combination of targets necessary to achieve the best efficacy. This approach can also resolve challenges in translation by inferring networks for different species and comparing differences in biology. Fundamentally, network inference enables a holistic view of the wealth of information in high dimensional data to provide a deeper understanding of biology.

Though network inference is a promising approach in principle, realizing this promise in practice depends upon the methods used. Reviews of benchmarking challenges using real and realistic simulated data [20] have shown that iterative feature selection methods outperform the alternatives, excluding methods using time series or knock-up/knock-down experiment data unavailable in clinical settings. One reason iterative feature selection methods are able to better identify true relationships is that, in comparison to simpler pair-wise or univariate methods such as correlation or mutual information scores, these methods model the multivariate effect of all predictors on a given response and thus capture interactions between predictors. Among iterative feature selection methods, GENIE3, a random forest based method [16], has shown the best individual performance overall particularly when tested on real data. This high performance can be understood from the strengths of random forest [3] as a tool for feature selection: it is able to identify nonlinear relationships and does not make assumptions about the distribution of the data.

The method we propose here is intended to retain the strengths shown by the GENIE3 algorithm (iterative feature selection with a nonlinear, nonparametric method) while addressing the threshold-dependence of these methods, as discussed above. Our method addresses this limitation by using a bespoke machine learning method for nonlinear, nonparametric feature selection [31] to identify sparse subsets of meaningful features. Rather than ranking all features as candidate predictors for a given response, this method excludes any features which do not improve prediction of the response. As a result, the method identifies a network with no additional need for thresholding. Additionally, we use this ‘subset-focused’ method in parallel with the weight-focused GENIE3 method to both identify a specific network from the data and accurately rank relationships within the network based on confidence. We name our implementation of the feature selection approach ‘DEKER’ for decomposed kernel regression, the core principle of the original method, and the network inference approach ‘DEKER-NET.’ We describe the methodology briefly in Methods and in complete detail in the ‘Appendix’, and illustrate the performance of DEKER-NET on the benchmarking dataset from the DREAM4 challenge [7] in ‘Results’. A C++ library implementation of the complete method is available at github.com/Merck/deker.

## Methods

DEKER-NET incorporates several novel improvements to enable subset-focused network inference. The core of the methodology is the feature selection method, which was originally described by Sun et al. [31]. This method is described briefly in ‘Nonlinear feature selection’, below. To best leverage this method for network inference, we implement a version with a novel strategy for selecting hyperparameters, DEKER, described in the ‘Appendix’. Additionally, in the process of assembling the features inferred by DEKER for network inference in DEKER-NET, we use GENIE3’s approach to weight edges. The complementarity between DEKER-NET and GENIE3 improves on both subset selection by DEKER and edge weighting by random forest in GENIE3. This process is described in ‘Network inference using DEKER-NET’, with a complete algorithm in the ‘Appendix’.

### Nonlinear feature selection

The feature selection method originally described by Sun et al. [31] was chosen for DEKER-NET based on the characteristics of high-performing methods in the DREAM4 challenge [20]. Specifically, the method models nonlinear, multivariate relationships without assumptions about the underlying distribution of data. Additionally, the method selects a sparse subset of features, which as discussed above differentiates it from the weight-focused methods used in other network inference approaches.

Sun et al.’s feature selection method is based on the principles of kernel regression which uses the nearest neighbors of each sample or observation to predict response. The influence of each neighbor and the size of the neighborhood are defined by a kernel function. To illustrate, take a dataset $\mathbb {D} = \{(\mathbf {x}_i,y_i)\}^{N_s}$, with paired samples $\mathbf {x}_i$ and responses $y_i$, where $N_s$ is the number of samples. Each sample $\mathbf {x}_i$ is a vector of length $N_j$, the number of features, where $\mathbf {x}_i \in \mathbb {R}^{N_j}$ and $y_i \in \mathbb {R}$. A held-out response $y_*$ is predicted using its sample $x_*$ and the other samples and responses in the dataset using the kernel function *f*.

1 $$\begin{aligned}&\hat{y}_* = \frac{\sum ^{N_s}_{i = 1} f(\mathbf {x}_*,\mathbf {x}_i) y_i}{\sum ^{N_s}_{i = 1} f(\mathbf {x}_*,\mathbf {x}_i)} \end{aligned}$$

A common choice of kernel function *f* is the squared exponential kernel, which introduces a characteristic lengthscale hyperparameter $l_f$ that controls the size of the neighborhood used for predicting each sample.

2 $$\begin{aligned} f(\mathbf {x}_*,\mathbf {x}_i) = \mathrm {exp}\left(- \frac{\Vert \mathbf {x}_*-\mathbf {x}_i \Vert _2}{2 l_f^2}\right) \end{aligned}$$

Kernel regression can be used for feature selection by adding weights $\mathbf {w} \in \mathbb {R}^{N_j}$ which scale each feature in $x_{i,j}$, adjusting the relative contribution of each feature to the prediction or removing the feature entirely when $w_j = 0$,

3 $$\begin{aligned} \hat{y}_* = \frac{\sum ^{N_s}_{i = 1} f(\mathbf {w} \circ \mathbf {x}_*,\mathbf {w} \circ \mathbf {x}_i) y_i}{\sum ^{N_s}_{i = 1} f(\mathbf {w} \circ \mathbf {x}_*,\mathbf {w} \circ \mathbf {x}_i)} \end{aligned}$$

where $\circ$ is the Hadamard operator for element-wise multiplication between vectors. With the weights introduced, the feature selection problem can be stated as finding the weights that minimize the prediction error between the true held-out response $y_*$ and the prediction $\hat{y}_*$. For example the RGS algorithm [24], a specific implementation of kernel regression for feature selection, uses sum of squares for prediction error and holds out each response in the dataset (leave one out cross-validation, LOOCV).

4 $$\begin{aligned} \begin{aligned} \min _{\mathbf {w}} \quad&\sum ^{N_s}_{j = 1} \frac{1}{2}\left( y_j - \frac{\sum _{i \in \{1 \dots N_s \setminus j \} } f(\mathbf {w} \circ \mathbf {x}_i,\mathbf {w} \circ \mathbf {x}_j) y_i}{\sum _{i \in \{1 \dots N_s \setminus j\}} f(\mathbf {w} \circ \mathbf {x}_i,\mathbf {w} \circ \mathbf {x}_j)}\right) ^2 \\ \text {s.t.} \quad&\mathbf {w} \ge 0 \end{aligned} \end{aligned}$$

Sun et al. observe however that this formulation of the feature selection problem is very difficult to solve numerically as the minimization problem has many local minima and as a result the ideal performance of the algorithm cannot be realized.

As an alternative, Sun et al. decompose the kernel regression problem into a series of classification problems with a quasi-convex objective function which can be reliably solved for the global minimum. The core idea is that any regression problem can be re-interpreted as a joint set of classification problems. Specifically, a set of thresholds $\mathbf {t} \in \mathbb {R}^{N_t}$ is defined to divide the samples of the dataset $\mathbb {D}$ into two sets. Sun et al. use every response in the dataset as a threshold, as will we, though it is worth noting that fewer thresholds (ex. every other response, every third, etc.) could be used to speed computation at the cost of some performance. For a given threshold value $t_k$, samples are divided into subsets $\mathbb {T}_{< t_k}$ and $\mathbb {T}_{\ge t_k}$ based on their responses $y_i$.

5 $$\begin{aligned} \begin{aligned} \mathbb {T}_{< t_k}&= \{i \vert y_i < t_k, i \in \{1 \dots N_S \} \} \\ \mathbb {T}_{\ge t_k}&= \{ i \vert y_i \ge t_k, i \in \{1 \dots N_S \} \} \end{aligned} \end{aligned}$$

Samples can then be classified in the same way response values are predicted in kernel regression: using the kernel-weighted distance of each sample’s predictors to the predictors in each subset. The performance of the classifier is then assessed based on the difference between the kernel-weighted distance to the correct subset and the incorrect subset based on the sample’s true response, which is called the margin *m*.

6 $$\begin{aligned} \begin{aligned} m(i,k)&= y^c_{i,k} \left( \sum _{j \in \mathbb {T}_{< t_k}} \Bigg[\frac{f(\mathbf {w} \circ \mathbf {x}_i,\mathbf {w} \circ \mathbf {x}_j)}{\sum _{i \in \mathbb {T}_{< t_k}} f(\mathbf {w} \circ \mathbf {x}_i,\mathbf {w} \circ \mathbf {x}_j)} \right. \\&\qquad \qquad \qquad \qquad * |\mathbf {w} \circ (\mathbf {x}_i-\mathbf {x}_j) | \Bigg] \\&\qquad - \sum _{j \in \mathbb {T}_{\ge t_k}} \Bigg[ \frac{f(\mathbf {w} \circ \mathbf {x}_i,\mathbf {w} \circ \mathbf {x}_j)}{\sum _{i \in \mathbb {T}_{\ge t_m}} f(\mathbf {w} \circ \mathbf {x}_i,\mathbf {w} \circ \mathbf {x}_j)} \\&\qquad \qquad \qquad \qquad * |\mathbf {w} \circ (\mathbf {x}_i-\mathbf {x}_j) |\Bigg]\Bigg) \\ y^{c}_{i,k}&= {\left\{ \begin{array}{ll}\! -1 &{} y_i < t_k \\ 1 &{} y_i \ge t_k \end{array}\right. } \end{aligned} \end{aligned}$$

This formulation can be used to construct a minimization problem equivalent to Eq. (4). Sun et al. specifically use L1 distance to simplify the calculation of derivatives for minimization. Additionally, Sun et al. include a penalty based on feature weights $\mathbf {w}$, as is done in LASSO regression [32] to impose sparsity. This penalty ensures that only a subset of predictive features with unique predictive power is selected, excluding uninformative features or features that are correlated to true predictors and not uniquely informative. The penalty also adds another hyperparameter $\lambda$, which controls the strength of the penalty. The resulting objective function to be minimized is

7 $$\begin{aligned} \min _{\mathbf {w}}&\sum ^{N_s}_{i=1} \sum _{k=1}^{N_t} m(i,k) + \lambda \sum ^{N_J}_{j=1} w_j \\ \text {s.t.} \quad&\mathbf {w} \ge 0 \end{aligned}$$

In their original description of the method [31], Sun et al. demonstrate superior performance to many other modern machine learning methods, including RGS [31].

When implementing this method, Sun et al. do not suggest an approach for selecting values of the two introduced hyperparameters, the characteristic lengthscale in the kernel function $l_f$ and penalty weight $\lambda$. Hyperparameters are distinguished from ordinary parameters learned from data, such as the weights $\mathbf {w}$, as they control the learning process itself and must be determined separately to prevent overfitting. Sun et al. test a range of hyperparameter values in their benchmarking exercise and conclude that the influence of the values on the features selected is relatively minor, recommending that values are fixed a priori. However we found while benchmarking using the DREAM4 challenge data [7] the features selected by the algorithm are strongly influenced by the values of the hyperparameters and fixing the values a priori can lead to very poor performance. The DREAM4 challenge data represent a larger and more varied set of test cases compared to Sun et al.’s original benchmarking, including many relationships between predictor and response that are weak relative to noise. An appropriate strategy for selecting hyperparameters thus appears critical to the method’s performance on realistic data for network inference.

A standard approach to selecting hyperparameter values would be to use nested cross-validation in which data are held out from the learning process and used to estimate how well selected features would predict response values on new data, repeated for combinations of hyperparameter values until an optimum value is found [6]. We found however that the feature selection is fairly unstable in this case. For example, using ten-fold cross validation in which a unique tenth of the dataset is held out and the algorithm selects features on the ten overlapping but unique datasets for a given value of $l_f$ and $\lambda$, five folds may select one feature, three folds may select two features, and two folds may select five features. Worse still, we observed that the features selected on each fold with 90% of the total data often do not reflect the features selected when the algorithm is run with all of the data. These liabilities substantially reduced the performance of Sun et al.’s feature selection in our tests.

To address these liabilities, we use another approach to estimate the performance of selected features on new data and identify optimum values of hyperparameters. Specifically, we calculate Bayesian Information Criterion (BIC) [30] values for prediction performance by estimating the degrees of freedom of a trained model of selected features [9] and using Platt scaling [19, 26] to condition margin values into probabilities suitable for calculating the log-likelihood of the predictions (details in ‘Appendix’). Additionally, we define a null BIC value to compare performance against. If a model’s BIC is higher than the null value, the features selected are uninformative and are excluded from the inferred network.

A complete description of the methodology is provided in the ‘Appendix’, including additional notes on practical considerations for our implementation of Sun et al.’s methodology, DEKER. Our implementation of DEKER is available at github.com/Merck/deker and can be used as either a stand-alone executable intended for use in a high-performance computing environment, or as a header-only C++ library for development.

### Network inference using DEKER-NET

**Fig. 1:**
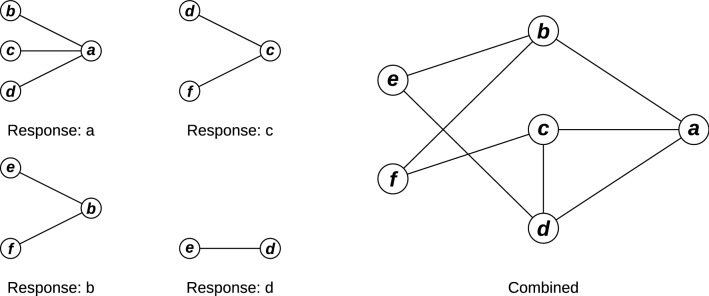
Illustrating network assembly. On the left, iterations of feature selection for responses **a**–**d** are visualized as networks. On the right is the network obtained by combining the relationships identified in the iterations of feature selection. Networks are illustrated without edge directionality for consistency; see ‘Results’ main text regarding directed vs. undirected network inference

In network inference by iterative feature selection, an iteration of feature selection is done to identify the predictors of each unique feature in the dataset. The results of a single iteration of feature selection can be visualized as a simple network: the response and each identified predictor is represented by a vertex, and edges are directed from each predictor to the response. The combined output of all feature selection iterations is the union of each of these simple networks: each feature in the dataset is represented by a vertex, which are connected by edges indicating other features which a given feature was either found to predict or found to be predicted by using feature selection. A simple example of this assembly is illustrated in Fig. 1. This network can also be represented as an adjacency matrix, $\mathbf {A}$, where each element $A_{i,j}$ indicates whether feature *j* is a predictor of feature *i* ($A_{i,j} > 0$) or not ($A_{i,j}=0$).

Weight-focused network inference methods such as GENIE3 [16] output a network where all vertices are connected, or $A_{i,j} > 0$ for all *i*, *j*. A sparse network can only by identified from this weighting by excluding edges with weights below an unknown threshold. When the true network structure is known the performance of these methods is assessed based on the error rates of the range of networks constructed by eliminating edges from the lowest to highest weight. In particular, the precision, or ratio of true positive to total inferred edges, and recall, or ratio of true positive to total edges in the true network, are used to capture the trade off between including the most true edges and excluding the most false edges. For a weight-focused method, ideal performance is realized if there exists a weight threshold which separates all true edges from all false edges. Even in this ideal case however, there is no method for identifying the optimal threshold in the practical case where the true network structure is unknown.

By contrast, iteratively applying Sun et al.’s feature selection outputs a single sparse network, where only a minority of possible edges $A_{i,j}$ are weighted. In practice, while weight-focused methods are concerned with a ranking of possible networks, a subset-focused method is concerned with identifying a single network structure for analysis and interpretation. There is a trade off, however. It is still desirable to rank edges within the network inferred by DEKER-NET, however the non-zero weights produced by Sun et al.’s feature selection are not comparable across models. As the network is constructed by combining all models, these weights cannot be used to rank edges.

Fortunately, weight-focused and subset-focused methods are complementary. For DEKER-NET, we use DEKER to select the sparse network while taking the edge weights from GENIE3. Compared to using GENIE3 alone, DEKER-NET explicitly determines which edges to exclude, including the majority of GENIE3’s low weighted edges as well as some highly weighted false-positive edges. Additionally, DEKER sometimes includes edges which are ranked very low by GENIE3. These edges are also excluded from the final network, further capitalizing on the complementarity between DEKER’s sparse feature selection and GENIE3’s weighting. As a result, DEKER-NET can often realize the best performance of GENIE3 while identifying a sparse, interpretable network. An algorithm detailing the combination of methods and network construction is provided in the ‘Appendix’.

## Results

To illustrate the performance of DEKER-NET, we use a common benchmarking approach on simulated multifactorial data. In multifactorial data, each sample is simulated from a network with multiple unknown perturbations relative to a baseline. These data imitate the available clinical data: each sampled patient has multiple unique, unknown differences in underlying biology relative to the others. Inferring networks from this type of data is much more challenging compared to precisely controlled gene knock-up or knock-down data, or time-series data with multiple observations from the same patient. Nevertheless, this type of data is easiest to acquire from real patients and best represents the real use-case of this methodology.

Alongside DEKER-NET, we test ‘Correlation’ network inference, which uses the absolute value of the Pearson correlation coefficient to weight edges, and two of the highest performing network inference methods, TIGRESS [10] and GENIE3 [16]. The Correlation approach is presented to illustrate worst-case performance, as this is the simplest to implement and most prone to errors. Results for TIGRESS and GENIE3 were generated using the R packages with default values for each [15, 33], with the exception of the K parameter for GENIE3 which was changed to ‘all’ as the authors suggest in [16].

While all methods (except Correlation) are able to infer the directionality of edges, in practice the results are relatively poor when applied to multifactorial data compared to undirected inference [16]. Therefore in these tests we consider only performance on identifying undirected structure. For example, if the underlying true network has the relationship $a \rightarrow b$, we accept either the edge $a \leftarrow b$ or $a \rightarrow b$ as true edges, using highest weight each directional edge to define the undirected edge $a \leftrightarrow b$.

As discussed in ‘Methods’, a common approach to assessing performance of network inference methods uses precision (ratio of true edges inferred to total edges inferred) and recall (ratio of true edges inferred to total edges in true network). In particular, each point on a precision-recall curve is generated by iteratively removing the lowest-ranked edge and recalculating the precision and recall values of the resulting network for any given network inference method. The most common error we observe during benchmarking is an indirect relationship inferred as a direct relationship. To illustrate such an indirect effect error, take a triplet such as $a \leftrightarrow b \leftrightarrow c$. If the network inference method infers a relationship between $a \leftrightarrow c$, it is an indirect effect error. These errors have relatively little effect on the structure of the inferred network, however: *a* and *c* are closely related in the true network structure, and an erroneous edge has a minor effect on network topology and the resulting interpretation. By contrast, an error connecting two features which are very distant in the true network has a much more serious effect both on the topology of the inferred network and the biological interpretation. Equating indirect effect errors and more serious errors therefore gives an overly pessimistic view of the performance of these methods. A more optimistic version of the standard precision-recall curve can be identified by excluding these indirect effect errors: for a given point, recall values remains the same while precision may improve. We visualize this by plotting our precision-recall curves as an area bounded by an upper curve corresponding to the precision and recall values calculated omitting indirect effect errors and a lower curve corresponding to precision and recall values including indirect effect errors.

In the first subsection, we apply DEKER-NET to the ‘Dialogue for Reverse Engineering Assessments and Methods’ initiative’s fourth (DREAM4) challenge on in silico multifactorial data [7]. DREAM4 challenge data have previously been used to assess the performance of a number of a variety of different network inference methods and provides a common reference point for comparison. In the second subsection, we perform the same tests on datasets with an increasing numbers of samples simulated from the DREAM4 challenge networks [29] to illustrate the sensitivity of our method to data availability and set expectations for performance on datasets of different sizes.

### Benchmarking with DREAM4 challenge data

**Fig. 2:**
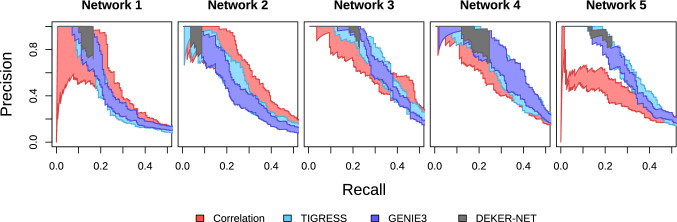
Precision-recall curves for DREAM4 in silico multifactorial challenge data show DEKER-NET is generally nearly equal to or better than GENIE3 performance. Precision-recall curves are constructed from the series of networks generated by iteratively removing the lowest-weighted edge of each network and calculating precision (ratio of true inferred edges to total inferred eges) and recall (ratio of true inferred edges to total edges in the true network). Each method is plotted as an area bounded by an upper curve corresponding to the precision and recall values calculated omitting indirect effect errors and a lower curve corresponding to precision and recall values including indirect effect errors; see ‘Results’ main text for details on indirect effect errors. An ideal precision-recall curve would maintain a values of 1 for precision accross all values of recall. One curve (method) is considered superior to another if its curve has a higher precision across a relevant range of recall values (Color figure online)

In Fig. 2 we show the precision-recall curves for each of the five networks in the DREAM4 in silico multifactorial dataset with 100 features and 100 samples per network. We plot both the curve for the raw precision values and precision ignoring indirect effect errors. Note that while the weight-focused Correlation, GENIE3, and TIGRESS methods have curves spanning the full range of recall values, the curve for our subset-focused DEKER-NET method ends abruptly with precision values at or greater than  70%. As discussed in ‘Methods’, this is by design as the goal of DEKER-NET is to identify a sparse network, while the weight-focused methods rank all possible relationships. DEKER-NET generally shows a minor improvement over other methods in terms of precision and recall, including GENIE3.

**Fig. 3:**
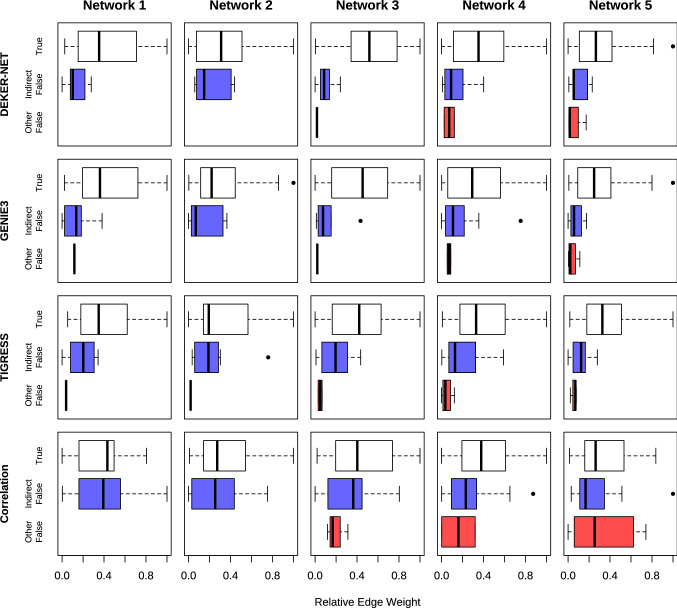
DEKER-NET shows nearly equal or better separation between true and false edges by relative edge weight compared to other methods for DREAM4 in silico multifactorial challenge data. For DEKER, GENEI3, and TIGRESS the majority of false edges are indirect false edges which have a lower impact on network structure than other false edges. Edge weights are rescaled to [0, 1] to enable comparison between methods with differing intrinsic scales. Distribution of edge weights for each edge type are illustrated as box-and-whiskers, with the whiskers corresponding to the minimum (left) and maximum (right), the edges of the box corresponding to the first quartile (left) and third quartile (right), the central line indicating the median value, and extreme values plotted as individual points

In Fig. 3 we show the distribution of edge weights among true edges, indirect error edges, and other error edges. This figure illustrates how effectively each method separates true edges from error edges by edge weight. For DEKER-NET, GENIE3, and TIGRESS, the majority of errors are indirect, and other errors separate fairly well from true or indirect error edges by weight. Overall, DEKER-NET offers a marginal improvement in separating errors from true edges by weight over GENIE3 by eliminating a few incorrect edges that would otherwise be highly weighted by GENIE3 while otherwise retaining the same weights and edge ranking.

**Fig. 4:**
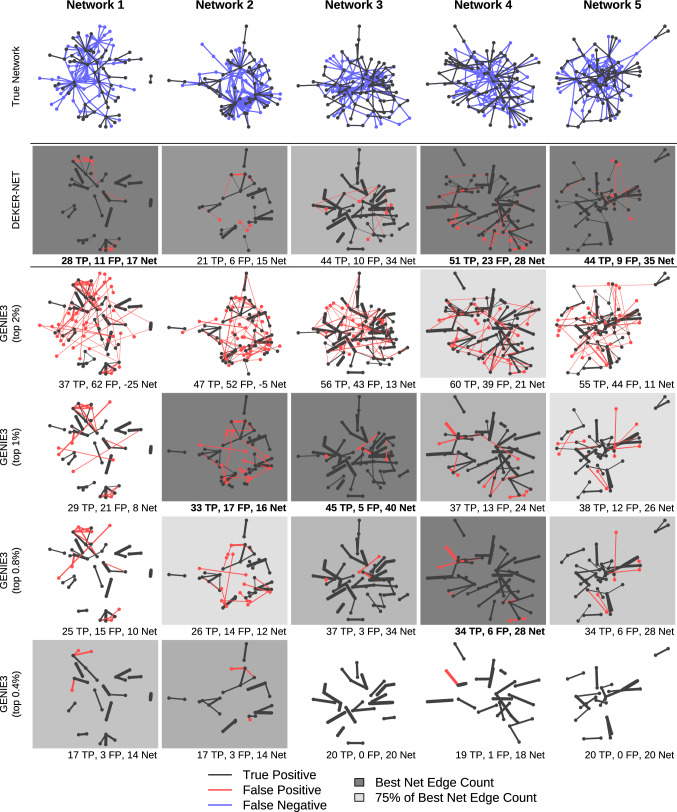
DEKER-NET performs network inference nearly equal to or better than GENIE3’s best performing edge weight threshold for all DREAM4 in silico multifactorial challenge datasets. GENIE3’s performance depends on the threshold value selected, which is not known in practice and varies substantially even between the similar DREAM4 networks. Threshold values used in this figure are based on commonly used literature values (see text): the top 2%, 1%, 0.8%, and 0.4% of edges. Inferred networks are shaded based on performance relative to the best performing inferred network for each dataset, where the best performing network has the darkest background shading. Networks with less than or equal to 66% of the best performing network’s performance are unshaded. Edge width corresponds to edge weight, with wider edges ranked more highly and assigned higher confidence by inference methods. Edges present in the true network structure but not identified by any inference method are false negatives shown in the true network (top row), while correctly identified edges in the true structure (by at least one method) are true positives shown in both the true network and corresponding inference method network. Edges not present in the true network structure but inferred by a given method are false positives shown in the corresponding inference method network. The total number of true positives (TP), false positives (FP), and net positive edges (TP-FP) are given for each inferred network (Color figure online)

To illustrate real-world performance we plot the network inferred by DEKER-NET alongside the networks inferred by GENIE3 in Fig. 4. For GENIE3 network construction we use a range of thresholds also used in the literature [8, 14, 27, 34] ranging from the top 0.2% to top 2% of edges. The 0.2% threshold was omitted from our figures, as this threshold universally resulted in too few edges to be considered. Additional figures for comparison to TIGRESS and correlation, both of whigh perform generally worse than GENIE3, are available in Supplemental Figures. To compare networks we use net edge count (true positive edges minus false positive edges) as a heuristic, which we will refer to as ‘net’ performance. This heuristic captures the dual criteria of increasing network size and reducing the number of errors, where adding a true positive has the same weight as removing a false positive. For ranking purposes this heuristic is equivalent to a reweighted mean of precision and recall, such that adding true positives and removing false positives have an equal effect on that mean.

Comparing the networks in Fig. 4 based on net positive edges, DEKER-NET generally performs as well as or better than GENIE3’s best thresholding scenario. DEKER-NET also performs better over all across datasets than any single threshold, as the performance for specific thresholds for GENIE3 is inconsistent based on dataset. Specifically, DEKER-NET has a higher net performance than GENIE3 for networks 1 and 5 for all thresholds, nearly equal net performance to GENIE3’s best threshold for networks 2 and 4, and equal net performance to GENIE3’s second best threshold for network 3 as illustrated by the shading in Fig. 4. Additionally, net performance across thresholding scenarios for GENIE3 illustrates the difficulty in selecting a threshold: for network 1, the top 0.4% of edges was best, for networks 2 and 3 the top 1% of edges was best, and for networks 4 and 5 the top 0.8% of edges was best. Considering that there is no way to gauge error rates to select the best threshold when the underlying network is unknown, this variance in performance across thresholds makes inference of novel networks unreliable for GENIE3. By contrast, we have demonstrated that DEKER-NET is able to reliably achieve equivalent or better performance without thresholding, enabling more effective inference of novel networks.

### Benchmarking with increasing sample size

To understand how the performance of DEKER-NET depends on the amount of data used we use the GeneNetWeaver [21, 29] program to simulate additional data sets equivalent to the 100-sample DREAM4 in silico multifactorial datasets tested in the previous section. We used the settings provided in the program that were used to generate the original DREAM4 data. The same five networks used in the DREAM4 challenge were used to simulate the dynamics for our test datasets. Using GeneNetWeaver, we generated 400 additional samples per network, and tested the algorithms as in the previous section using draws of 100, 200, and 400 samples from those generated in each network. We do note generally worse performance for all methods on the DREAM4-like data compared to the DREAM4 benchmarks, which may be due to small differences in software version or variance in the simulated data from run to run.

**Fig. 5:**
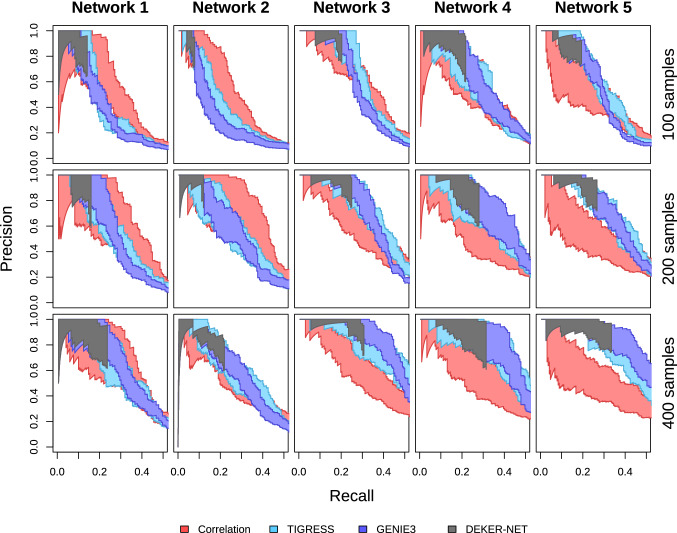
Precision-recall values improve as the number of samples increase for DREAM4-like datasets. Precision-recall curves are constructed from the series of networks generated by iteratively removing the lowest-weighted edge of each network and calculating precision (ratio of true inferred edges to total inferred edges) and recall (ratio of true inferred edges to total edges in the true network). Each method is plotted as an area, with the upper curve corresponding to the precision and recall values calculated omitting indirect effect errors and the lower curve corresponding to precision and recall values including indirect effect errors. See ‘Results’ main text for details on indirect effect errors (Color figure online)

**Fig. 6:**
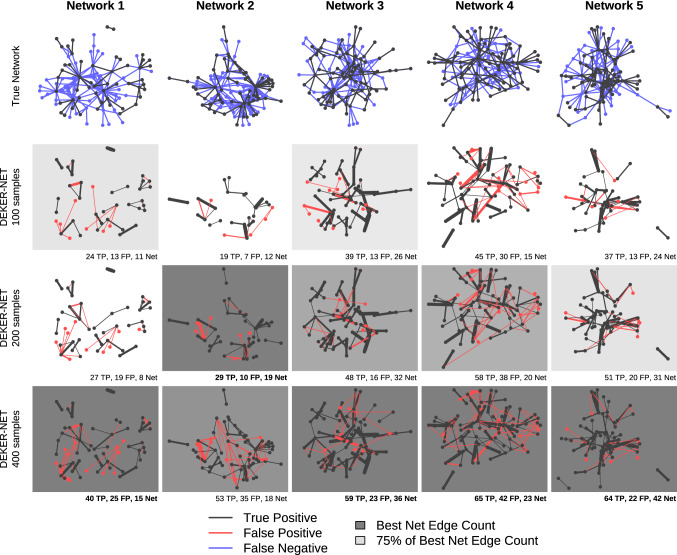
DEKER-NET identifies larger networks with generally increasing performance as the number of samples increase for DREAM4-like datasets. Inferred networks are shaded based on performance relative to the best performing inferred network for each dataset, where the best performing network has the darkest shading. Networks with less than or equal to 66% of the best performing network’s performance are unshaded. Edge width corresponds to edge weight, with wider edges ranked more highly and assigned higher confidence by inference methods. Edges present in the true network structure but not identified by any inference method are false negatives shown in the true network (top row), while correctly identified edges in the true structure are true positives shown in both the true network and corresponding inference method network. Edges not present in the true network structure but inferred by a given method are false positives shown in the corresponding inference method network. The total number of true positives (TP), false positives (FP), and net positive edges (TP-FP) are given for each inference method network (Color figure online)

Precision-recall curves for inference with increasing sample size, shown in Fig. 5, demonstrate a modest increase in the maximum recall that each method achieves for given level of precision as sample size increases. The results suggest that, for the DREAM4 and DREAM4-like data, the majority of relationships that can realistically be inferred are found with a relatively modest amount of data. This is likely due to the inherent difficulty of network inference on multifactorial data. We also note that the precision of the full subset of edges identified by DEKER-NET is generally consistent at $\ge 60\%$, worse than observed on the DREAM4 data, though non-indirect errors occurred at a very low rate. Figure 6 further illustrates that each doubling of sample size has a less than proportional effect on the network subset that DEKER-NET infers; four times the data generally yields less than twice the number of true edges, with comparable precision.

Overall, the performance of DEKER-NET as sample size increases suggests that for datatypes and biological networks similar to the gene regulatory network data that GeneNetWeaver emulates, there are diminishing returns in increasing sample size and that 100–200 samples may be an ideal target depending on cost constraints in generating data. Sampling from different populations may be more impactful than increasing sample size in a given population, however this was not possible to test in the given benchmarking framework. Additionally, it is unknown how the size and complexity of the true network to be inferred may affect the number of samples needed for inference.

## Discussion

Prior to DEKER-NET, network inference methods were primarily limited by their focus on weighting and ranking all possible relationships, which produces a dense, uninterpretable network structure. Interpretation of these dense networks requires heuristic tuning of a weight threshold to produce a sparse network, a process which is error-prone and not well-defined in the literature. DEKER-NET addresses this limitation by identifying a sparse, interpretable network directly. Furthermore, DEKER-NET incorporates the best-performing weight-focused method, GENIE3 [16] enabling both selection of a sparse network and effective ranking of edges within that network. Through the synergy between feature selection approaches, DEKER-NET maintains the lowest error rates while selecting an interpretable sparse network, addressing an important limitation of earlier methods.

The tests in ‘Results’ focus on realistic scenarios in terms of data quality and quantity, setting expectations for real-world performance of DEKER-NET. These tests use multifactorial data, which best imitates the type of data received from patients with many unknown sources of variation in biology, simulated using a framework intended to imitate a range of realistic network structure and levels of noise [29]. As a result, the networks inferred are incomplete, typically capturing only  20% of the true structure of the biological network used to simulate the data. Regardless, the information gained about this portion of network structure is useful: for networks inferred by DEKER-NET in our tests, 7 of every 10 identified relationships is a true, mechanistic biological relationship. Additionally, the incorrectly inferred relationships are primarily indirect effect errors, which link closely related features that share a common relationship. These indirect effect errors have minimal impact on the overall structure of the network in comparison with more serious errors linking features more distant in the true network topology.

Given the challenges presented by multifactorial data, we believe DEKER-NET is most useful as a hypothesis generating tool. The identified network structure is a reliable, albeit incomplete, description of biological pathways present in the dataset. These pathways can also be considered a focused set of mechanistic hypotheses best supported by the available data, and can direct experimental follow-up to the most relevant biology.

Another important consideration for the application of network inference methods are the range of types of biological data (’omes) to be analyzed, and particularly the fusion of data from distinct domains and technologies into multi-omic datasets. The DREAM4 benchmarking data are intended to imitate gene expression or transcriptomic data, and further work is needed to understand how methods perform on different data types or combinations thereof. In principle, the machine learning methods upon which DEKER-NET is built are robust to different data types, as it does not rely on any assumptions about the underlying distribution of data or the relationships between features, and works with continuous and categorical data. Similarly, biologically plausible results have been shown from applying GENIE3 to real multi-omic data [11, 12]. It remains important to further test performance across data types.

The applications of network inference are broad. In general, the DEKER-NET network inference methodology is a tool for gaining insight into biology. It can be used to identify targets or biomarkers when applied to data with specific endpoints of interest, or to understand translation when applied to data from multiple different species. Most importantly, it supports these critical questions with clear, precise hypotheses and anchors efforts in drug discovery and development to biology in a repeatable and objective way.

### Supplementary Information

Below is the link to the electronic supplementary material.

Supplementary file1 (PDF 320 kb)

## Appendix

Here we first review the original implementation of the feature selection method proposed by Sun et al. [31]. In addition we propose a simple practical improvement for solving the fixed-point iteration problem that arises, and discuss considerations for optimizing hyperparameters using Akaike or Bayesian Information Criterion (AIC, BIC). Additionally, we detail the use of a null model and the resulting AIC or BIC value to identify cases in which no features should be selected. The algorithm implementation details for both the feature selection method and the overall network inference method are summarized at the end.

### Decomposed kernel regression

As discussed in ‘Methods’, Sun et al. decompose the kernel regression problem into a series of classification problems which creates a quasi-convex optimization problem which can be reliably solved for the global minimum by reformulating the kernel regression problem into a series of classification problems. A range of classification thresholds $\mathbf {t} \in \mathbb {R}^{N_t}$ are defined to divide the samples of the dataset $\mathbb {D}$ into subsets $\mathbb {T}_{< t_m}$ and $\mathbb {T}_{\ge t_m}$ based on their responses $y_i$. An unknown response $y_*$ can then be classified based on the distance function *d* of the unknown sample’s predictors $\mathbf {x}_*$ to its nearest neighbor in each subset:

8 $$\begin{aligned} \mathbb {T}_{< t_m}&= \{i \vert y_i< t_m, i \in \{1 \dots N_S \} \} \nonumber \\ \mathbb {T}_{\ge t_m}&= \{ i \vert y_i \ge t_m, i \in \{1 \dots N_S \} \} \nonumber \\ y_*< t_m&\Leftrightarrow \min _{i \in \mathbb {T}_{< t_m}} d(\mathbf {w} \circ \mathbf {x}_*, \mathbf {w} \circ \mathbf {x}_i) \nonumber \\&\quad\quad< \min _{i \in \mathbb {T}_{\ge t_m}} d(\mathbf {w} \circ \mathbf {x}_*, \mathbf {w} \circ \mathbf {x}_i) \nonumber \\ y_* \ge t_m&\Leftrightarrow \min _{i \in \mathbb {T}_{< t_m}} d(\mathbf {w} \circ \mathbf {x}_*, \mathbf {w} \circ \mathbf {x}_i) \nonumber \\&\quad\quad > \min _{i \in \mathbb {T}_{\ge t_m}} d(\mathbf {w} \circ \mathbf {x}_*, \mathbf {w} \circ \mathbf {x}_i) \end{aligned}$$

With this formulation of the problem, the goal is to find the weights $\mathbf {w}$ which maximize correct prediction of response $y_*$ classification labels, $y^{c}_{*,m}$, for all thresholds $t_m$ in $\mathbf {t}$. Response classification labels are represented as

9 $$\begin{aligned} y^{c}_{*,m} = {\left\{ \begin{array}{ll}\! -1 &{} y_* < t_m \\ 1 &{} y_* \ge t_m \end{array}\right. } \end{aligned}$$

Given the basis of this classification on the distance between nearest neighbors, classification performance can be best achieved by maximizing the distance to the wrong subset and minimizing the distance to the correct subset, or maximizing the margin $\Delta d$:

10 $$\begin{aligned}\Delta d_{*,m} = y^{c}_{*,m}&\left( \min _{i \in \mathbb {T}_{< t_m}} d(\mathbf {w} \circ \mathbf {x}_*, \right. \nonumber \mathbf {w} \circ \mathbf {x}_i) \\&\quad- \left. \min _{i \in \mathbb {T}_{\ge t_m}} d(\mathbf {w} \circ \mathbf {x}_*, \mathbf {w} \circ \mathbf {x}_i) \right) \end{aligned}$$

Unfortunately, when solving with respect to the weights $\mathbf {w}$, the nearest neighbor $\mathbf {x}_i$ in each subset is unknown and needs to be approximated and updated. Sun et al. use a kernel function *f* in a manner analogous to kernel regression, giving an estimate of the margin $\Delta \hat{d}$, with fixed weights $\mathbf {u}$ equal to the intial weights $\mathbf {w}$ at each step of the iterative updating process:

11 $$\begin{aligned} \begin{aligned}\Delta \hat{d}_{*,m} = y^{c}_{*,m} & \left( \sum _{i \in \mathbb {T}_{< t_m}}\right. \Bigg[ \frac{f(\mathbf {u} \circ \mathbf {x}_*, \mathbf {u} \circ \mathbf {x}_i)}{\sum _{i \in \mathbb {T}_{< t_m}} f(\mathbf {u} \circ \mathbf {x}_*, \mathbf {u} \circ \mathbf {x}_i)} \\&\quad \quad \quad \quad * d(\mathbf {w} \circ \mathbf {x}_*, \mathbf {w} \circ \mathbf {x}_i)\Bigg]\\&- \sum _{i \in \mathbb {T}_{\ge t_m}} \Bigg[\frac{f(\mathbf {u} \circ \mathbf {x}_*, \mathbf {u} \circ \mathbf {x}_i)}{\sum _{i \in \mathbb {T}_{\ge t_m}} f(\mathbf {u} \circ \mathbf {x}_*, \mathbf {u} \circ \mathbf {x}_i)}\\&\quad \quad \quad \quad * d(\mathbf {w} \circ \mathbf {x}_*, \mathbf {w} \circ \mathbf {x}_i)\Bigg]\Bigg) \end{aligned} \end{aligned}$$

Many choices of kernel function are possible, and we will use the commonly employed squared exponential or radial basis function kernel,

12 $$\begin{aligned}&f(\mathbf {x}_*,\mathbf {x}_i) \nonumber = \mathrm {exp}\left({- \frac{\Vert \mathbf {x}_*-\mathbf {x}_i \Vert _2}{2 l_f^2}}\right) \end{aligned}$$

where $l_f$ is the characteristic length scale, a hyperparameter controlling the rate of exponential decay with distance between each point. Next, by choosing the L1 distance for the distance function *d*, the effects of each individual predictor feature are now separated and the margin represented as a linear function of the weights $\mathbf {w}$:

13 $$\begin{aligned} \begin{aligned} \Delta \hat{d}_{*,m}&= \sum _{j=1}^{N_j} w_j y^{c}_{*,m}\left( \sum _{i \in \mathbb {T}_{< t_m}} \frac{f(\mathbf {u} \circ \mathbf {x}_*, \mathbf {u} \circ \mathbf {x}_i)}{\sum _{i \in \mathbb {T}_{< t_m}} f(\mathbf {u} \circ \mathbf {x}_*, \mathbf {u} \circ \mathbf {x}_i)} |x_{*,j}-x_{i,j} |\right. \\&\left.\quad\quad\quad\quad\quad\quad\quad - \sum _{i \in \mathbb {T}_{\ge t_m}} \frac{f(\mathbf {u} \circ \mathbf {x}_*, \mathbf {u} \circ \mathbf {x}_i)}{\sum _{i \in \mathbb {T}_{\ge t_m}} f(\mathbf {u} \circ \mathbf {x}_*, \mathbf {u} \circ \mathbf {x}_i)} |x_{*,j}-x_{i,j} |\right) \\&\overset{\Delta }{=} \sum _{j=1}^{N_J} w_j z_{*,m,j} \end{aligned} \end{aligned}$$

To ensure that the smallest set of predictor features are selected, sparsity is imposed on the solutions in two ways. First, rather than use the raw margin $\Delta \hat{d}$ we use a loss function based on hinge loss, which constrains maximum performance such that increases in the classification margin stop improving the loss function once a critical threshold is reached. This helps to reduce the number of features selected by preventing extraneous or correlated features from improving the margin $\Delta \hat{d}$ after the classifier has reached a sufficiently high performance. The specific loss function we use is a differentiable approximation of hinge loss based on Huber loss [4], which has a quadratically smoothed elbow to enable differentiation of the function at the elbow,

14 $$\begin{aligned} \begin{aligned} H(\Delta \hat{d})&= {\left\{ \begin{array}{ll}\! 0 &{} \Delta \hat{d}> 1 + h\\ \frac{(1+h-\Delta \hat{d})^2}{4h} &{} 1-h \le \Delta \hat{d} \le 1+h \\ 1-\Delta \hat{d} &{} \Delta \hat{d}< 1 + h \end{array}\right. } \\ \frac{\mathrm {d} H}{\mathrm {d} \Delta \hat{d}}\left( \Delta \hat{d} \right)&= {\left\{ \begin{array}{ll} 0 &{} \Delta \hat{d} > 1 + h\\ \frac{\Delta \hat{d}-h-1}{2h} &{} 1-h \le \Delta \hat{d} \le 1+h\\ -1 &{} \Delta \hat{d} < 1 + h \end{array}\right. } \end{aligned} \end{aligned}$$

where *h* is a parameter controlling the size of the elbow of the hinge loss function. In this formulation, increases in the margin $$\Delta \hat{d}$$ have no effect on the loss function above a value of 1, which is set arbitrarily: changing this value will only change the absolute, but not relative, values of the weights $$\mathbf 
{w}$$. The number of features selected is further constrained using regularization, specifically an L1 penalty as in LASSO regression [32], is also used to limit the number of features selected. This penalty, $$\lambda \sum ^{N_j}_{j=1} w_j$$, introduces a hyperparameter $$\lambda$$ which controls the trade-off between predictive performance and the number of features selected.

The final optimization problem is then constructed as in Eq.(4) by leave one out cross validation: each sample is held out and predicted from all other samples in the dataset, and the loss function is minimized with the regularization penalty across all classification thresholds $\mathbf {t}$

15 $$\begin{aligned} \begin{aligned} \min _{\mathbf {w}} \quad&L(\mathbf {w}) = \sum _{k=1}^{N_s} \sum _{m=1}^{N_t} H\left( \sum _{j=1}^{N_J} w_j z_{k,m,j} \right) + \lambda \sum ^{N_j}_{j=1} w_j \\ \text {s.t.} \quad&\mathbf {w} \ge 0 \end{aligned} \end{aligned}$$

where $z_{k,m,j}$ is calculated as in Eq. (13), with sample *k* held out

16 $$\begin{aligned} \begin{aligned} z_{k,m,j} = y^{c}_{k,m}&\left( \sum _{i \in \mathbb {T}_{< t_m} \setminus k} \frac{f(\mathbf {u} \circ \mathbf {x}_k, \mathbf {u} \circ \mathbf {x}_i)}{\sum _{i \in \mathbb {T}_{< t_m} \setminus k} f(\mathbf {u} \circ \mathbf {x}_k, \mathbf {u} \circ \mathbf {x}_i)} |x_{k,j}-x_{i,j} |\right. \\&\left. - \sum _{i \in \mathbb {T}_{\ge t_m} \setminus k} \frac{f(\mathbf {u} \circ \mathbf {x}_k, \mathbf {u} \circ \mathbf {x}_i)}{\sum _{i \in \mathbb {T}_{\ge t_m} \setminus k} f(\mathbf {u} \circ \mathbf {x}_k, \mathbf {u} \circ \mathbf {x}_i)} |x_{k,j}-x_{i,j} |\right) \end{aligned} \end{aligned}$$

Sun et al. convert the constrained optimization problem (15) to an unconstrained optimization problem by substituting $v_j^2 = w_j$ for all $i \in {1 \dots N_j}$,

17 $$\begin{aligned}\min _{\mathbf {v}} \quad L(\mathbf {v}) =& \sum _{k=1}^{N_s} \sum _{m = 1}^{N_t} \nonumber H\left( \sum _{j=1}^{N_J} v^2_j z_{k,m,j} \right) \\& + \lambda \sum ^{N_j}_{j=1} v^2_j \end{aligned}$$

where the derivative for each $v_i \in \mathbf {v}$ is

18 $$\begin{aligned}&\mathrm {d} L/\mathrm {d} v_i = 2 v_i \left[ \sum _{k=1}^{N_s} \sum _{m=1}^{N_t} \frac{\mathrm {d} H}{\mathrm {d} \Delta \hat{d}}\left( \sum _{j=1}^{N_j} v^2_j z_{k,m,j}\right) z_{k,m,i} + \lambda \right] \end{aligned}$$

allowing the minima to be easily found by unconstrained optimization techniques such as LBFGS, rprop, or similar [28, 35]. As noted previously, the problem must be solved by iteratively updating the initial weights $\mathbf {u}$,

19 $$\begin{aligned} \mathbf {u}(n+1) \overset{\Delta }{=} \mathbf {v}^2(n) \end{aligned}$$

where *n* is the fixed point iteration number. Convergence thus occurs when $\mathbf {u}(n+1) = \mathbf {v}^2(n)$ (to within a specified numerical tolerance). Having described the original method as proposed by Sun et al., we now explain a number of modifications which we have used to improve the performance of the method and increase interpretability in the following sections.

### Accelerating fixed-point iteration

We observe that the fixed point iteration between weights $\mathbf {u}$ and $\mathbf {w}$ is often unnecessarily slow and vulnerable to cycles. To remedy this, we use Steffensen’s method generalized for systems [13]. In practice this method uses three consecutive iterations of weights $\mathbf {u}(n+1)$, $\mathbf {u}(n+2)$, $\mathbf {u}(n+3)$ to produce an accelerated update, $\mathbf {u}^*(n+1)$,

20 $$\begin{aligned}&\mathbf {u}^*(n+1) \overset{\Delta }{=} \mathbf {u}(n) - \Delta \mathbf {U}(n) \nonumber \\&\quad * \left( \Delta ^2 \mathbf {U}(n)\right) ^{-1} \left( \mathbf {u}(n+1)-\mathbf {u}(n)\right) \end{aligned}$$

where $\Delta \mathbf {U}(n)$ and $\Delta ^2 \mathbf {U}(n)$ are matrices of the change in weights *u* accross updates,

21 $$\begin{aligned} \begin{aligned} \Delta \mathbf {U}(n)&= \left( \mathbf {u}(n+1)-\mathbf {u}(n),\right. \\&\quad\quad\quad \left. \mathbf {u}(n+2)-\mathbf {u}(n+1)\right) \\ \Delta ^2 \mathbf {U}(n)&= \left( \mathbf {u}(n+2)-\mathbf {u}(n+1),\right. \\&\quad\quad\quad \left. \mathbf {u}(n+3)-\mathbf {u}(n+2)\right) \end{aligned} \end{aligned}$$

While the generalized Steffen’s method requires additional computational effort for each update $\mathbf {u}^*(n+1)$, in practice we found that the acceleration to the fixed point solution and ability to avoid cycles dramatically reduces the overall effort in solving the feature selection problem.

### Optimizing hyperparameters

Finally, a major consideration in the solution of the feature selection problem in Eq. (17) is the selection of hyperparameters, specifically the regularization parameter $\lambda$ and characteristic lengthscale parameter $l_f$ in Eq. (12). Hyperparameters are distinguished from ordinary parameters as values which control the process of learning a model from data, and therefore cannot be identified during the model fitting process. While Sun et al. found the hyperparameters to have little influence in their tests, we found substantial influence of these parameters on the predictor feature subsets selected in our benchmarking. As discussed in ‘Methods’, this is undesirable due to the computational burden and the nature of feature selection.

Alternatively, we use an estimate of out-of-sample prediction error from the subset of predictor features selected using the entire dataset such as the Akaike or Bayesian Information Criterion (AIC, BIC) [1, 30] to select hyperparameters. We will use BIC, as we find it gives better performance from our benchmarking by tending to select fewer features, although AIC can be easily substituted based on the user’s preference. To calculate BIC we need to determine the model fit in terms of likelihood and the degrees of freedom of the fit model. We note that for these calculations we will not be holding samples out as is done when training the weights $\mathbf {w}$ to estimate out-of-sample predictor error, as this is handled separately by the penalty to model complexity.

To determine the likelihood of the model fit for BIC we use Platt scaling [19, 26]: we fit a logistic regression model to the raw classifier values to produce calibrated probability values which can be used to determine the likelihood of the fit. First, a matrix of classifier values without samples held-out $\mathbf {q}$ is calculated for each sample *k* (rows) and classification threshold $t_m$ (columns), similar to Eq. (16).

22 $$\begin{aligned} \begin{aligned}q_{k,m}(l_f) = \sum _{j=1}^{N_j} w_j & \Bigg( \sum _{i \in \mathbb {T}_{< t_m}} \Bigg[ \frac{f(\mathbf {u} \circ \mathbf {x}_k, \mathbf {u} \circ \mathbf {x}_i)}{\sum _{i \in \mathbb {T}_{< t_m}} f(\mathbf {u} \circ \mathbf {x}_k, \mathbf {u} \circ \mathbf {x}_i)} \\&\quad\quad\quad\quad *|x_{k,j}-x_{i,j}|\Bigg]\\& - \sum _{i \in \mathbb {T}_{\ge t_m}} \Bigg[\frac{f(\mathbf {u} \circ \mathbf {x}_k, \mathbf {u} \circ \mathbf {x}_i)}{\sum _{i \in \mathbb {T}_{\ge t_m}} f(\mathbf {u} \circ \mathbf {x}_k, \mathbf {u} \circ \mathbf {x}_i)} \\&\quad\quad\quad\quad * |x_{k,j}-x_{i,j} |\Bigg]\Bigg) \end{aligned} \end{aligned}$$

Then, using the true class values $\mathbf {y}^c$ rescaled to 0, 1 ($\mathbf {y}^p$),

23 $$\begin{aligned} y^p_{k,m} = \frac{y^c_{k,m}+1}{2} \end{aligned}$$

the values $\mathbf {q}$ are fit to a logistic function by maximizing the negative log likelihood.

24 $$\begin{aligned} \begin{aligned} \hat{y}^p_{k,m}(l_f)&= \frac{1}{1+e^{A q_{k,m}(l_f) + B}} \\ \hat{L}(l_f)&= \max _{A,B} \quad - \sum _{k,m}^{N_s,N_t} y^p_{k,m} \bigg[\ln {(\hat{y}^p_{k,m}(l_f))} \\&\quad\quad\quad\quad\quad\quad + (1-y^p_{k,m})\ln {(1-\hat{y}^p_{k,m}(l_f))}\bigg] \end{aligned} \end{aligned}$$

The maximum value of the negative log likelihood $\hat{L}(l_f)$ can then be used to calculate BIC by adding a penalty proportional to the degrees of freedom, as described below.

Determining the degrees of freedom of the fit model is more challenging given the structure of the fit model. An approach that we found effective is to formulate the model in terms of a hat matrix,

25 $$\begin{aligned} \hat{\mathbf {y}}^p(l_f) = \mathbf {H} \mathbf {y}^p \end{aligned}$$

where the true labels $\mathbf {y}^p$ and predictions $\hat{\mathbf {y}}^p$ are each matrices with number of samples $N_s$ rows and number of classification thresholds $N_t$ columns, and the hat matrix $\mathbf {H}$ is a matrix with $N_s$ rows and columns which represents the fit model. Hastie and Tibshirani [9] have shown that the trace of the hat matrix is an appropriate definition of the effective degrees of freedom of the fit model. Identifying this hat matrix from the model is somewhat challenging, however it can be readily approximated from the true labels and predictions using

26 $$\begin{aligned}&\hat{\mathbf {H}} = \mathbf {H} \mathbf {y}^p \mathbf {y}^{p+}\\&\quad =\hat{\mathbf {y}}^p(l_f) \mathbf {y}^{p+} \end{aligned}$$

where $\mathbf {y}^{p+}$ is the Moore–Penrose inverse (pseudoinverse) of the matrix of predicted values, and $\mathbf {H} = \hat{\mathbf {H}}$ if and only if the right product of the label matrix and its pseudo inverse is the identity matrix $\mathbf {y}^p \mathbf {y}^{p+} = \mathbf {I}$ [2, 23, 25]. Additionally, this calculation can fail when the matrix of true labels is not invertible. For the typical case where the value of each sample (in the rows of the matrix) is used as a classification threshold (in columns), the threshold dividing the dataset using the lowest response value should be discarded, as by definition all response values will be greater than or equal to this response value and no classification is possible. This produces a matrix of true labels with linearly independent columns, and the pseudoinverse can be calculated.

27 $$\begin{aligned} \mathbf {y}^{p+} = (\mathbf {y}^{pT} \mathbf {y}^p)^{-1} \mathbf {y}^{pT} \end{aligned}$$

In this case, the pseudoinverse of the label matrix is the left inverse of the label matrix, and the product $\mathbf {y}^p \mathbf {y}^{p+}$ is not the identity matrix. In practice however, particularly when each sample has a unique response value, it is very nearly the identity matrix, with the only difference a 0 for the first diagonal value of the matrix. When multiple samples share the same response value, the product of $\mathbf {y}^p \mathbf {y}^{p+}$ will have non-zero off-diagonal values for each element where the pair of samples corresponding to a given row and column have the same response value. In these cases, the non-zero off-diagonal elements and diagonal elements will be 1 divided by the number of samples sharing the response value, and all rows and columns still sum to 1. To account for this in our estimate, we use the sum of any value with a non-zero element in the product of $\mathbf {y}^p \mathbf {y}^{p+}$ rather than the trace of $\hat{\mathbf {H}}$. We find this estimate to give good performance in our benchmarking results.

Finally, taking the negative log likelihood $\hat{L}(l_f)$ from Eq. (23) together with the degrees of freedom definition, we can calculate the BIC for each fit of the regularization $\lambda$ and characteristic lengthscale $l_f$ hyperparameters.

28 $$\begin{aligned} \mathrm {BIC}(l_f) = \ln {(N_s)} N_t \mathrm {tr}(\hat{\mathbf {y}}^p(l_f) \mathbf {y}^{p+}) + 2 \hat{L}(l_f) \end{aligned}$$

Note that we multiply the degrees of freedom by the number of classification thresholds $N_t$ as we are duplicating the model that many times when calculating $\hat{L}$. In addition to this calculation, it is very useful to determine the BIC of a null model to compare against. In the context of feature selection, this is particularly important as it allows us to determine that no features should be selected when the best fit model does not perform better than the null. Considering a null model in the form of a hat matrix, a natural candidate is a matrix which averages all labels, predicting for each $\hat{y}^p_{k,m}$ the relative frequency of labels $y^p_{k,m}$ equal to 1. When our labels $\mathbf {y}^p$ are treated as a matrix with each column predicted independently, an averaging matrix will generate predictions based on the relative frequency of labels for each classification problem. We observed during benchmarking that this null model is too restrictive: many models identifying true features were found to have higher BIC values than this null model. An alternative, equivalent formulation of the null model considers the labels $\mathbf {y}^p$ and predictions $\hat{\mathbf {y}}^p$ as column vectors by concatenating the columns of all classification threshold problems into one column vector with length $N_t N_s$. In this context, the hat matrix is likewise a matrix with $N_t N_s$ rows and columns. In the case of the fit model, the matrix $\hat{\mathbf {H}}$ solved in Eq. (26) is repeated down the diagonal with all other elements set to 0. In this context we consider another averaging matrix as the null model, where all elements are equal to $\frac{1}{N_t N_s}$. This matrix takes the average of all labels, generating a prediction for all classification problems which is equal to the relative frequency of (1) valued labels (as labels are either 0 or 1). This allows us to calculate the null BIC value,

29 $$\begin{aligned} \begin{aligned} T_{y^p}&= \sum _{k,m}^{N_s,N_t} y^p_{k,m} \\ \mathrm {BIC}_0&= \ln {(N_s)} + 2 [\ln (\frac{T_{y^p}}{N_t N_s}) T_{y^p} \\&+ \ln (1-\frac{T_{y^p}}{N_t N_s}) (N_t N_s - T_{y^p})] \end{aligned} \end{aligned}$$

given that the degrees of freedom will always equal 1 by definition for the null hat matrix.

It is now relatively straightforward to optimize the regularization $\lambda$ and characteristic lengthscale $l_f$ hyperparameters by minimizing the BIC using a derivative-free global optimization algorithm. In particular we find that a Bayesian optimization [22] framework such as the limbo library [5] is a very effective choice, as the tradeoff between computation time and robust exploration of the parameter space can be explicitly controlled. Furthermore, we also observe that the characteristic lengthscale $l_f$ parameter (or other kernel-specific hyperparameters, if a different function is used) can be optimized during the fixed point iterations for feature weights $\mathbf {v}^2$. When updating the matrix $\mathbf {z}$ (Eq. (16)) with new weights $\mathbf {u}$, we optimize for the kernel hyperparameters before solving for the next iteration of weights $\mathbf {v}^2$.

30 $$\begin{aligned} \begin{aligned} \hat{l}_f&= {{\,\mathrm{arg\,min}\,}}_{l_f} \quad \mathrm {BIC}(l_f) \\&= {{\,\mathrm{arg\,min}\,}}_{l_f} \quad \ln {(N_s)} N_t \mathrm {tr}(\hat{\mathbf {y}}^p(l_f) \mathbf {y}^{p+})\\&\quad\quad\quad\quad\quad\quad\quad\quad + 2 \hat{L}(l_f) \end{aligned} \end{aligned}$$

The optimal characteristic lengthscale $\hat{l}_f$ is then used to update the $\mathbf {z}$ matrix before solving for feature weights in Eq. (17). Separating the optimization of the parameters in this way substantially saves on computational effort, as optimizing in the dimension of kernel parameters only requires recalculation of the matrix $\mathbf {z}$ and BIC value, rather than optimization of feature weights $\mathbf {v}^2$.

In the case of the squared exponential kernel, optimizing the characteristic lengthscale $l_f$ hyperparameter is a convex problem: increasing $l_f$ causes prediction from a larger neighborhood of points, reducing the degrees of freedom while increasing the negative log likelihood due to a poorer fit, while decreasing $l_f$ has the opposite effect, with the lowest BIC value corresponding to the optimal trade-off. This problem can be readily solved by local gradient-free optimization algorithms, however long plateaus in the BIC value when the characteristic lengthscale $l_f$ reaches a critical upper or lower value can cause convergence issues or waste computation time. Fortunately, these critical values can be approximated with simple calculations and a bounded local optimization algorithm used to easily optimize the hyperparameter. The lower critical value occurs when the response of each sample is predicted from only a single other point, as reducing the characteristic lengthscale further cannot select fewer points. As each sample will always be closest to itself in the calculation for BIC with a distance of 0, we are then concerned with the value for $l_f$ at which the kernel function value for the smallest non-zero distance is very large. Similarly, the upper critical value is determined by the point at which the kernel function value for the largest distance is very small, as at this point the response of a sample will be predicted from all other samples. Thus we can easily solve for these bounds using the equation for the kernel Eq. (12),

31 $$\begin{aligned} \begin{aligned} d_{min} =&\min _{i,j} \quad \Vert \mathbf {u} \circ (\mathbf {x}_i-\mathbf {x}_j) \Vert _2 \\&\text {s.t.} \Vert \mathbf {x}_i-\mathbf {x}_j \Vert _2 > 0 \\ l_{f \text {min}} =&\sqrt{\frac{-d_{min}}{2 \ln 1/\delta }} \\ d_{max} =&\max _{i,j} \quad \Vert \mathbf {u} \circ (\mathbf {x}_i-\mathbf {x}_j) \Vert _2 \\ l_{f \text {max}} =&\sqrt{\frac{-d_{max}}{2 \ln \delta }} \end{aligned} \end{aligned}$$

where $\delta$ is a sufficiently small value (we use $10^{-5}$).

We outline the complete algorithm for fitting the model for a given regularization $\lambda$ value in Algorithm 1, and the network inference procedure in Algorithm 2.
