# Supplementary material for: From complex data to biological insight: ‘DEKER’ feature selection and network inference
Source: J Pharmacokinet Pharmacodyn. 2021 Nov 17;49(1):81–99. doi: 10.1007/s10928-021-09792-7 (PMC8837529; doi:10.1007/s10928-021-09792-7)
Supplement: Supplementary file 1 — Supplementary file1 (PDF 320 kb) [file 10928_2021_9792_MOESM1_ESM.pdf]

Supplemental Figures

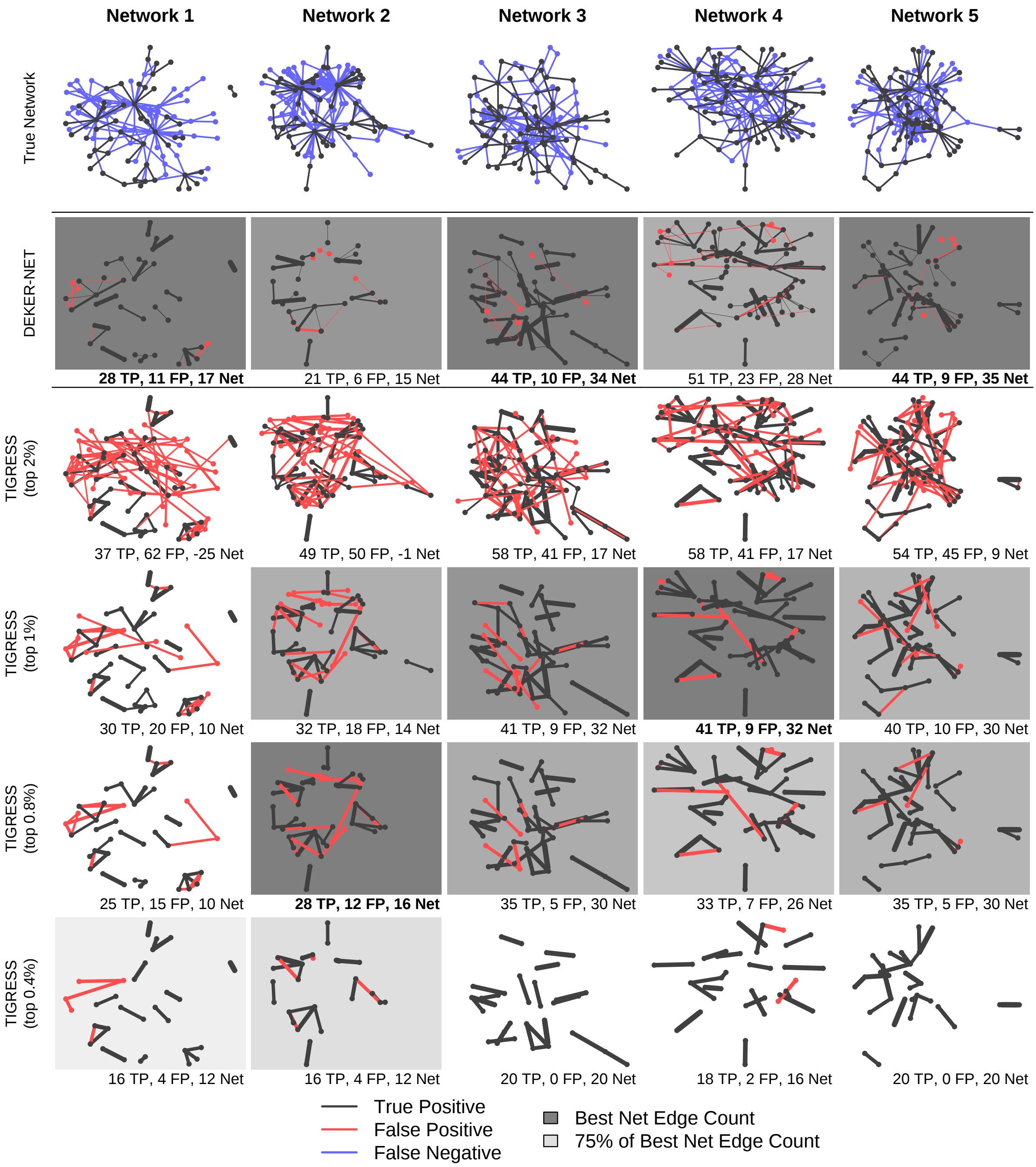

Fig. 1 DEKER-NET performs network inference nearly equal to or better than TIGRESS's best performing edge weight threshold for all DREAM4 *in silico* multifactorial challenge datasets. TIGRESS's performance depends on the threshold value selected, which is not known in practice and varies substantially even between the similar DREAM4 networks. Threshold values used in this figure are based on commonly used literature values (see text): the top 2%, 1%, 0.8%, and 0.4% of edges. Inferred networks are shaded based on performance relative to the best performing inferred network for each dataset, where the best performing network has the darkest background shading. Networks with less than or equal to 66% of the best performing network's performance are unshaded. Edge width corresponds to edge weight, with wider edges ranked more highly and assigned higher confidence by inference methods. Edges present in the true network structure but not identified by any inference method are false negatives shown in the true network (top row), while correctly identified edges in the true structure (by at least one method) are true positives shown in both the true network and corresponding inference method network. Edges not present in the true network structure but inferred by a given method are false positives shown in the corresponding inference method network. The total number of true positives (TP), false positives (FP), and net positive edges (TP-FP) are given for each network.

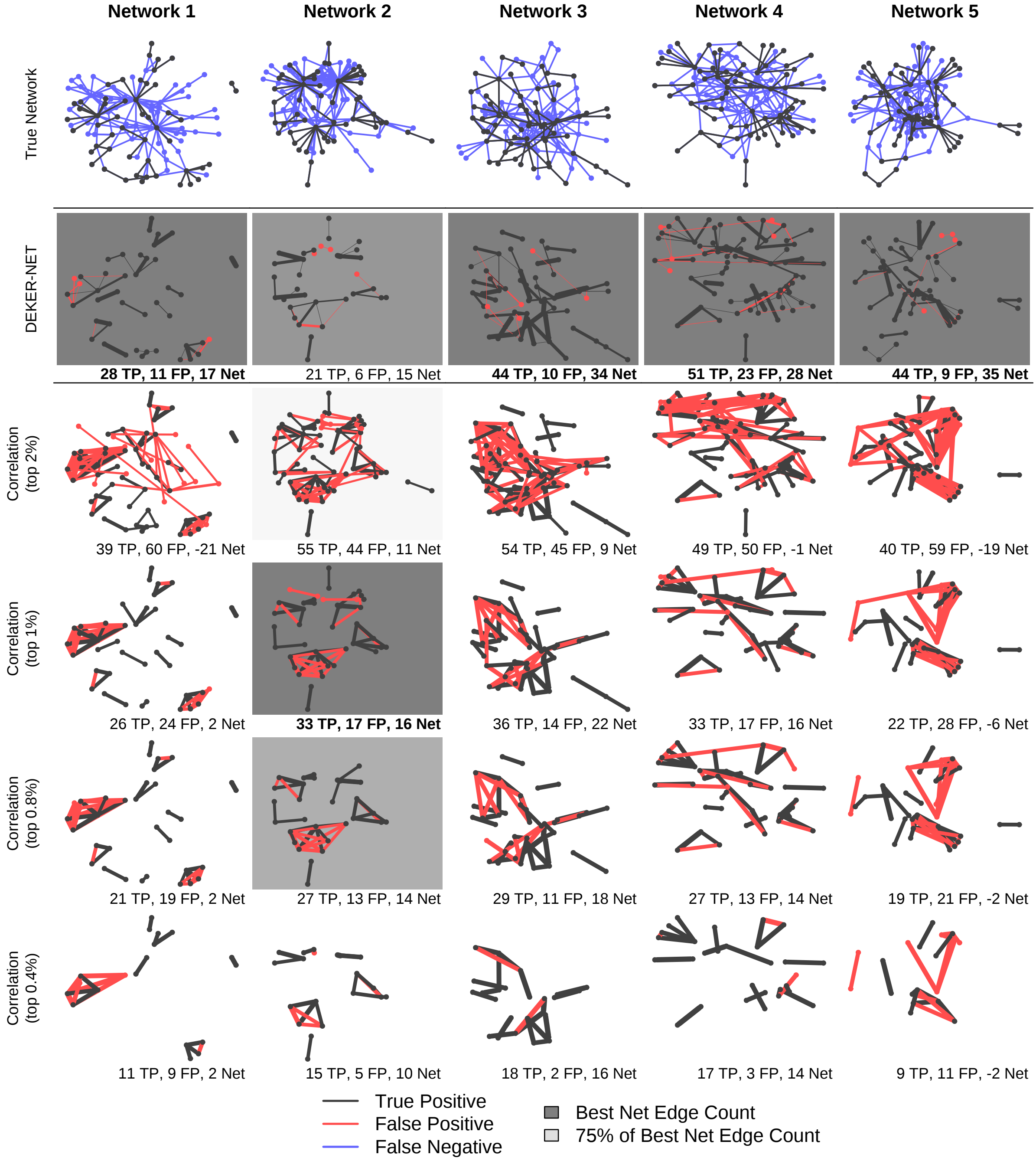

Fig. 2 DEKER-NET performs network inference nearly equal to or better than correlation's best performing edge weight threshold for all DREAM4 *in silico* multifactorial challenge datasets. Correlation's performance depends on the threshold value selected, which is not known in practice and varies substantially even between the similar DREAM4 networks. Threshold values used in this figure are based on commonly used literature values (see text): the top 2%, 1%, 0.8%, and 0.4% of edges. Inferred networks are shaded based on performance relative to the best performing inferred network for each dataset, where the best performing network has the darkest background shading. Networks with less than or equal to 66% of the best performing network's performance are unshaded. Edge width corresponds to edge weight, with wider edges ranked more highly and assigned higher confidence by inference methods. Edges present in the true network structure but not identified by any inference method are false negatives shown in the true network (top row), while correctly identified edges in the true structure (by at least one method) are true positives shown in both the true network and corresponding inference method network. Edges not present in the true network structure but inferred by a given method are false positives shown in the corresponding inference method network. The total number of true positives (TP), false positives (FP), and net positive edges (TP-FP) are given for each network.
